# Characterization of Microbial Shifts during the Production and Ripening of Raw Ewe Milk-Derived Idiazabal Cheese by High-Throughput Sequencing

**DOI:** 10.3390/biology11050769

**Published:** 2022-05-18

**Authors:** Gorka Santamarina-García, Igor Hernández, Gustavo Amores, Mailo Virto

**Affiliations:** Lactiker Research Group, Department of Biochemistry and Molecular Biology, Faculty of Pharmacy, University of the Basque Country (UPV/EHU), Paseo de la Universidad 7, 01006 Vitoria-Gasteiz, Spain; igor.hernandezo@ehu.eus (I.H.); gustavo.amores@ehu.eus (G.A.)

**Keywords:** cheese quality, ripening, microbiota, bacterial diversity, 16S rRNA sequencing, PCoA

## Abstract

**Simple Summary:**

Idiazabal is a traditional cheese produced from raw ewe milk in the Basque Country (Southwestern Europe). The sensory properties of raw milk cheeses have been attributed, among other factors, to microbial shifts that occur during the production and ripening processes. In this study, we used high-throughput sequencing technologies to investigate the microbiota of Latxa ewe raw milk and the dynamics during cheese production and ripening processes. The microbiota of raw milk was composed of lactic acid bacteria (LAB), environmental bacteria and non-desirable bacteria. Throughout the cheese making and ripening processes, the growth of LAB was promoted, whereas that of non-desirable and environmental bacteria was inhibited. Moreover, some genera not reported previously in raw ewe milk were detected and clear differences were observed in the bacterial composition of raw milk and cheese among producers, in relation to LAB and environmental or non-desirable bacteria, some of which could be attributed to the production of flavour related compounds.

**Abstract:**

In this study, we used high-throughput sequencing technologies (sequencing of V3–V4 hypervariable regions of 16S rRNA gene) to investigate for the first time the microbiota of Latxa ewe raw milk and the bacterial shifts that occur during the production and ripening of Idiazabal cheese. Results revealed several bacterial genera not reported previously in raw ewe milk and cheese, such as *Buttiauxella* and *Obesumbacterium*. Both the cheese making and ripening processes had a significant impact on bacterial communities. Overall, the growth of lactic acid bacteria (LAB) (*Lactococcus*, *Lactobacillus*, *Leuconostoc*, *Enterococcus*, *Streptococcus* and *Carnobacterium*) was promoted, whereas that of non-desirable and environmental bacteria was inhibited (such as *Pseudomonas* and *Clostridium*). However, considerable differences were observed among producers. It is noteworthy that the starter LAB (*Lactococcus*) predominated up to 30 or 60 days of ripening and then, the growth of non-starter LAB (*Lactobacillus*, *Leuconostoc*, *Enterococcus* and *Streptococcus*) was promoted. Moreover, in some cases, bacteria related to the production of volatile compounds (such as *Hafnia*, *Brevibacterium* and *Psychrobacter*) also showed notable abundance during the first few weeks of ripening. Overall, the results of this study enhance our understanding of microbial shifts that occur during the production and ripening of a raw ewe milk-derived cheese (Idiazabal), and could indicate that the practices adopted by producers have a great impact on the microbiota and final quality of this cheese.

## 1. Introduction

Idiazabal cheese is a semi-hard or hard cheese made exclusively from the raw milk of Latxa and/or Carranzana sheep, with a minimum ripening time of 60 days. Its production is located in the Basque Country (Southwestern Europe) and has a Protected Designation of Origin (PDO) [1]. Most of the producers attached to the Idiazabal PDO are small family dairies that lead the whole process, from livestock management to cheese making and final sales. Although Idiazabal cheese production is a strictly regulated process, producers may use different practices that may affect the characteristics of the final product. The most considerable differences in production practices are noticed in the management and feeding of the herd, leading to differences in milk quality [2]; in the use of artisanal or commercial rennet, or in the parameters selected during cheese making and ripening, since the Idiazabal PDO specifications establish ranges [3].

Idiazabal cheese, as other cheeses prepared from raw milk, has a richer and more intense aromatic profile compared with those produced from pasteurized milk [4,5]. Such interesting sensory properties of raw milk cheeses have previously been attributed, among other factors, to the complex dynamics of microbial composition during cheese making and ripening [5,6]. The quality of raw milk, use of starters and their intrinsic characteristics, type of rennet used and ripening time are some factors that determine the cheese microbiota [5,7,8,9]. The microbiota of milk has a diverse and complex composition, but it is mainly composed of lactic acid bacteria (LAB) [5,10]. In Idiazabal cheese, the most common LAB are *Lactococcus*, *Lactobacillus* and *Leuconostoc* [11,12]. These bacteria metabolize the lactose present in milk, generating lactic acid and other compounds, such as acetic acid, ethanol and diacetyl. These compounds, along with others produced during ripening, determine the sensory properties of cheese [13]. Although LAB are predominant, other low-abundance microorganisms are also part of the microbial ecosystem of cheese [10,14,15], and consequently contribute to the quality of the final product [16,17].

The characteristics of LAB and other microorganisms present in Idiazabal cheese have been described and related to its sensory properties in several studies [11,12,18,19]. However, these studies were performed 20 years ago using culture-dependent methods. Nowadays, high-throughput sequencing (HTS) technologies are used to monitor microbial communities in different fermented products [20,21,22], including cheese [23,24,25]. The HTS techniques allow the detection of a large number of bacteria, including those present in relatively small numbers [26,27,28,29], those present in a viable but non-cultivable state (VBNC) [30] and those not detected by other culture-dependent or independent methods [30,31,32,33,34].

The vast majority of studies on cheese focus on cheese produced from cow milk [24,35,36], and only a few studies have been carried out on cheese produced from the raw milk of ewe [37,38,39]. Moreover, little is known about the bacterial composition of raw ewe milk [40,41,42] and how it changes during cheese making and ripening processes [26,43,44].

Therefore, this study aimed to (1) characterize the bacterial communities of the raw milk of Latxa ewe; (2) analyse the effect of cheese making and ripening processes on bacterial populations; and (3) study the potential differences among producers producing the same type of cheese. To the best of our knowledge, no comprehensive metagenomic study has been conducted to date on raw ewe milk-derived cheeses. Moreover, although Idiazabal cheese has an internationally recognized PDO [1], no HTS studies have been performed to characterize its bacterial populations.

## 2. Materials and Methods

### 2.1. Milk and Cheese Sampling

To analyse the microbiota of Latxa ewe raw milk and Idiazabal cheeses, samples were collected from four artisanal Idiazabal PDO cheese producers (identified as A, B, C and D), whose dairies were situated in different geographic locations throughout the Basque Country. Milk was kept in refrigeration tanks before cheese making. Cheeses were produced from the collected milk samples, according to specifications issued by the Idiazabal Designation of Origin Regulatory Board [3], using Choozit MM 100 LYO 50 DCU (mixture of *Lactococcus lactis* subsp. *lactis*, *Lactococcus lactis* subsp. *cremoris* and *Lactococcus lactis* subsp. *lactis* biovar. *diacetylactis*) (DuPont NHIB Ibérica S.L., Barcelona, Spain) as the starter. Milk was coagulated using artisanal rennet prepared from the stomachs of Latxa lambs (extracted during the first month of lactation, cleaned, dried, salted and ground, as described previously [45]) or commercial rennet NATUREN^®^ 195 Premium (Chr. Hansen Holding A/S, Hørsholm, Denmark). Cheese ripening was carried out in chambers maintained at 8–14 °C temperature and 80–95% relative humidity. Cheeses were collected in duplicate at six time points during ripening (1, 7, 14, 30, 60 and 120 days). Therefore, a total of 4 raw milk samples and 48 cheese samples were analysed. Samples were collected and transported to the laboratory under refrigerated conditions (3 °C) for analysis.

### 2.2. DNA Extraction

DNA extraction was performed immediately after sample arrival, following the method described by Erkus et al. [46], with some modifications. To extract DNA from cheese samples, 10 g of each sample was suspended in 90 mL of 2% (*w*/*v*) sterile sodium citrate (pH 8.0), and homogenized in a stomacher (Masticator Basic 400; IUL Instruments, Königswinter, Germany) six times, each for 20 s ON and 10 s OFF. Then, 1.5 mL of the resulting suspension was centrifuged at 8000× *g* for 10 min at 4 °C, and the fat-containing supernatant was discarded. The obtained pellet was resuspended in 600 µL of sodium citrate, and centrifuged three times at 8000× *g* for 10 min at 4 °C. DNA was extracted with DNeasy Blood & Tissue Kit (Qiagen, Valencia, CA, USA), according to the manufacturer’s protocol. To extract DNA from milk samples, 10 mL of raw milk from each sample was processed as described above, however, without the need for homogenisation in the stomacher.

### 2.3. Library Preparation and Sequencing

HTS analysis was performed in the Sequencing and Genotyping Unit of the Genomic Facility/SGIker (supported by UPV/EHU, MICINN, GV/EJ, FSE) of the University of the Basque Country. The 16S rRNA gene library was prepared using Nextera XT DNA Library Preparation Kit (Illumina Inc., San Diego, CA, USA), according to the 16S rRNA gene metagenomics workflow of Illumina. The V3–V4 regions of the 16S rRNA gene were amplified by PCR (forward primer: 5′-TCGTCGGCAGCGTCAGATGTGTATAAGAGACAGCCTACGGGNGGCWGCAG-3′; reverse primer: 5′-GTCTCGTGGGCTCGGAGATGTGTATAAGAGACAGGACTACHVGGGTATCTAATCC-3′) as described by Klindworth et al. [47]. Then, 16S rRNA gene sequencing was performed on the Illumina MiSeq platform using the MiSeq Reagent Kit v3 (2 × 300 bp) (Illumina Inc.).

### 2.4. Bioinformatic Analysis

Quality filtering and trimming of raw reads were performed using the MiSeq Reporter software (Illumina), and taxonomic classification was performed using the MG-RAST web data analysis tool [48], based on the Silva SSU database [49]. Since the sequencing of most variable regions of the 16S rRNA gene is effective up to the genus level, and seldom discriminates among species adequately [50], the taxonomic classification was performed up to the genus rank. Rarefaction curves were also generated using MG-RAST.

### 2.5. Statistical Analysis

Relative bacterial abundance (%) was calculated based on the identified sequences, and three significant figures were used to express the results. The IBM SPSS statistical package version 26.0 (IBM SPSS Inc., Chicago, IL, USA, 2019) was used for data preparation and analysis. Mann–Whitney *U* test and Kruskal-Wallis analysis of variance with Bonferroni correction were performed using the SPSS package. The objective was to estimate differences in reads and operational taxonomic units (OTUs) between milk and cheese samples, and to analyse the influence of producer, cheese making and ripening time factors on bacterial phyla and genera abundance. To determine the direction and strength of correlations among the main bacterial genera, Spearman’s rank correlation coefficients were calculated using SPSS, and displayed as a heat map in RStudio version 1.3.959 and R version 3.6.3 [51] using the “gplots” package [52]. To analyse the effect of producer and ripening time factors on the abundance of the main bacterial genera, Permutational Multivariate Analysis of Variance (PERMANOVA) was computed in R using the “vegan” package [53]. Principal Component Analysis (PCA) of the main bacterial genera was performed using their log-transformed, when necessary, and Unit Variance scaled abundance data, and plotted using the SIMCA software (version 15.0.0.4783; Umetrics AB, Umeå, Sweden). The number of principal components (PCs) was determined by eigenvalues (greater than 1.5) and cross validation. The aim was to study microbial dynamics in cheeses according to producer and ripening time factors. An Orthogonal Partial Least Squares Discriminant Analysis (OPLS-DA) was performed in SIMCA to confirm whether microbial communities of samples differed according to the producer.

Alpha and beta diversity indices were calculated by taking into account the sequence abundance of all bacterial genera present in milk and cheese samples. Alpha diversity was assessed in R using different packages, depending on the objective: “tidyverse” package for data cleaning and preparation for analysis [54]; “BiodiversityR” package for calculating Shannon, Simpson, Inverse Simpson, Berger and Shannon evenness (Jevenness and Eevenness) diversity indices [55]; and “vegan” package for calculating Chao1 and ACE diversity indices. Significant differences among producers for each diversity index were analysed in SPSS using Kruskal-Wallis test. Beta diversity indices (Bray–Curtis and Jaccard dissimilarities) were calculated using the “vegan” package of R, and plotted into a Principal Coordinate Analysis (PCoA) model using the “APE” package of R [56].

## 3. Results and Discussion

### 3.1. Characteristics of 16S rRNA Gene Sequencing Data

A total of 10,798,992 16S rRNA gene sequences were obtained from Latxa ewe raw milk and Idiazabal cheese samples (*n* = 52), with an average sequence length of 348 ± 101 bp, mean GC content of 53 ± 5% and 10,388 OTUs. Altogether, 24 bacterial phyla, 209 families and 645 genera were identified. Further details of the reads, OTUs and number of identified phyla, families and/or genera are summarised in Table 1. The number of sequences obtained from cheese samples was significantly greater than those obtained from milk samples (*p* *≤* 0.001), although no significant differences were observed in the number of identified OTUs between the two sample types (*p >* 0.05). Moreover, both milk and cheese samples obtained from different producers showed significant dissimilarities in the number of reads (*p* *≤* 0.01) and identified OTUs (*p* *≤* 0.001), with producer A being clearly distinct from the other three producers. In general, the rarefaction curves showed a clear and strong stabilizing tendency (Figure S1), indicating sufficient sampling of microbial communities. Overall, this study reports a greater number of sequence reads, OTUs and taxonomic identifications in raw ewe milk and cheese than previous studies [14,38,39,44].

### 3.2. In-Depth Analysis of Microbial Shifts

#### 3.2.1. Bacterial Composition of the Raw Milk of Ewe

**Figure 1:**
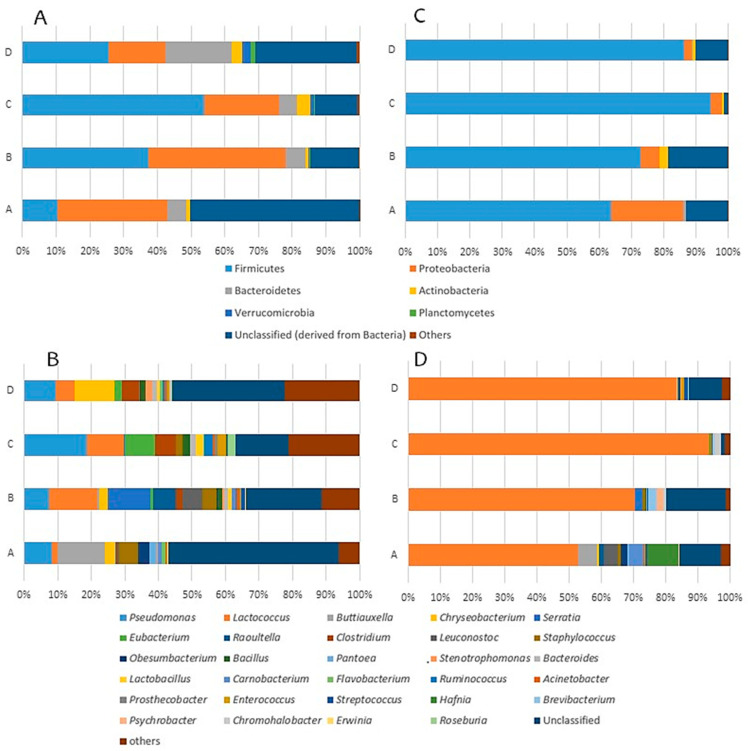
Relative abundance (%) of bacterial phyla and genera of Latxa ewe raw milk ((**A**,**B**), respectively) and 1-day-old ripened Idiazabal cheese samples ((**C**,**D**), respectively) produced by four producers (A, B, C, D).

Milk is an important source of microorganisms in cheese [5,57]. A total of 21 bacterial phyla, 165 families and 455 genera were identified in raw milk samples. At the phylum level (Figure 1A, Table S1), Firmicutes (10.5–54.1%) and Proteobacteria (16.9–40.7%) were the most dominant, followed by Bacteroidetes (5.44–19.6%). Other phyla, with abundances higher than 1%, were detected only in milk samples obtained from some producers: Actinobacteria and Verrucomicrobia in samples obtained from producer C (3.75% and 1.33%, respectively) and D (2.97% and 2.75%, respectively), and Planctomycetes in samples from producer D (1.28%). In general, the predominance of Firmicutes and Proteobacteria in raw ewe milk is consistent with previous studies [26,40,42], although differences in the abundance of each phylum have been reported among milk samples collected from different breeds [26,40,41,42]. However, raw milk samples of Latxa ewe were characterized by the high-level abundance of Bacteroidetes and a notable presence of Verrucomicrobia and Planctomycetes in comparison with milk collected from other breeds [26,40,42]. This indicates a differential characteristic of Latxa ewe raw milk used for Idiazabal cheese production. 

A total of 24 genera with abundance greater than 1% were identified, and 10 of these genera showed abundance higher than 5% (Figure 1B, Table S2). *Lactococcus* (1.64–14.5%), *Eubacterium* (0.0766–9.18%), *Clostridium* (0.183–6.09%), *Leuconostoc* (0–6.10%) and *Staphylococcus* (0.291–5.48%) were the most abundant genera within Firmicutes. Similarly, *Pseudomonas* (7.36–18.5%), *Buttiauxella* (0–14.1%), *Serratia* (0.0245–12.6%) and *Raoultella* (0–6.86%) showed the highest abundance within Proteobacteria, and *Chryseobacterium* (0–11.7%) within Bacteroidetes. Differences were observed among milk samples obtained from different producers (Table S2). While *Pseudomonas* and *Lactococcus* were identified as the main genera common to all analysed raw milk samples, the remaining genera were characteristic of each producer. The abundance of the rest of genera classified as “others” and unclassified sequences was remarkable (6.25–22.4% and 15.6–50.8%, respectively).

Differences observed in the microbial composition of milk samples at the phylum and genus levels among producers (Tables S1 and S2) could be caused by various factors such as differences in lactation stage, flock management and feeding, or sources of microorganisms, for instance, mammary gland diseases or microorganisms contaminating the teat surface, practices and materials employed during milking or dairy environment [5,10,15,41]. Moreover, these factors could explain the differences observed in bacterial communities between the raw milk of Latxa ewe and that of other ewe breeds [40,41,42].

The identified bacterial genera were divided into three groups: LAB, comprising genera previously classified as LAB [57]; environmental bacteria, including bacteria derived from the natural environment [58]; and non-desirable bacteria, containing genera exhibiting a pathogenic potential [59] or related to spoilage [60]. The LAB identified in this study included the genera *Lactococcus*, *Leuconostoc*, *Enterococcus*, *Lactobacillus*, *Carnobacterium* and *Streptococcus*. These gram-positive bacteria have frequently been identified in dairy products [57,61], and their presence in the raw milk of ewe breeds, other than Latxa, has been confirmed by HTS, albeit at different abundances [42,43,44].

The environmental bacterial genera identified in this study included *Obesumbacterium*, *Roseburia* and *Prosthecobacter*. These genera have been isolated from different natural sources, such as soil, fresh and salt water as well as animal and human gut [62,63,64]; however, to the best of our knowledge, no study has reported their presence in raw ewe milk.

The non-desirable bacterial genera identified in this study included *Pseudomonas*, *Clostridium*, *Staphylococcus* and *Bacillus*, which are widely known pathogens [59,65]. For instance, *Pseudomonas* is the most important psychrotrophic bacteria in raw milk, which may even predominate in refrigerated milk [10]. It comes from natural environment [66] and has been related to hygiene conditions [67,68]. Some species belonging to the genera *Buttiauxella*, *Serratia*, *Chryseobacterium*, *Eubacterium*, *Raoultella*, *Ruminococcus*, *Pantoea*, *Stenotrophomonas*, *Bacteroides*, *Flavobacterium* and *Acinetobacter* have also been described as opportunistic or as emerging pathogens [69,70,71,72,73,74,75,76,77,78,79]. Moreover, some of these genera, such as *Serratia* and *Clostridium*, are also related to milk spoilage, resulting in off-flavours [80] and to the cheese blowing defect because of CO_2_ production [81]. The presence of these bacteria in raw ewe milk has been reported only in a few studies [42,59,82,83]. To the best of our knowledge, the genera *Buttiauxella*, *Serratia*, *Eubacterium*, *Raoultella*, *Ruminococcus* and *Bacteroides* have not been identified in raw ewe milk so far.

#### 3.2.2. Bacterial Shifts during the Cheese Making Process

Next, the effect of the cheese making process, which encompasses all the production stages from milk to 1-day-old ripened cheeses, on microbiota was analysed. In this way, the bacterial composition of Latxa ewe raw milk and 1-day-old ripened Idiazabal cheese was compared. In 1-day-old ripened cheese samples, bacteria belonging to 19 phyla, 160 families and 450 genera were detected; thus, the number of identified bacterial families and genera were similar between 1-day-old ripened cheese and raw milk samples, but the number of bacterial phyla identified in cheese was less than that identified in raw milk. However, the cheese making process had a great impact on the abundance of bacterial communities (Figure 1C,D, Tables S1 and S2). At the phylum rank (Figure 1C, Table S1), the relative abundance of Firmicutes increased remarkably in 1-day-old ripened Idiazabal cheese samples (63.7–94.7%), while that of Proteobacteria decreased (2.61–22.4%), although remaining as the second most important phyla. In general, the abundances of the rest of phyla decreased, although the effect of the cheese making process was not statistically significant in all cases. The abundance of sequences classified as “others” was considerably reduced (<0.01%), and unidentified sequences accounted for lower, yet remarkable, abundance (1.21–18.6%). To date, very few HTS studies have analysed the effect of the cheese making process on bacterial communities in raw ewe milk cheeses [43,44], and even fewer at the phylum rank [26]. In comparison to raw ewe milk-derived cheeses, more HTS studies have been conducted on cow milk-derived cheeses [29,84]. De Pasquale et al. [26] have reported an increase in Firmicutes abundance and a decrease in Proteobacteria abundance in Canestrato Pugliese raw ewe milk-derived cheese, but the changes were more drastic than those observed in this study. No information could be found in the literature concerning the effect of the manufacturing process on the remaining phyla.

The effect of the cheese making process on the main bacterial genera is shown in Figure 1D and Table S2. Within LAB, *Lactococcus* was the most abundant genus in 1-day-old ripened cheese samples collected from all producers (52.5–93.2%), although a notably lower abundance was observed for producer A. The effect of the cheese making process on the remaining genera in the LAB group varied with the producer. The abundance of *Lactobacillus* decreased in cheese samples collected from all producers, except producer A (<0.200% in all producers); abundances of *Leuconostoc* and *Carnobacterium* were higher in producer A samples (4.48% and 4.40%, respectively); abundances of *Streptococcus* and *Enterococcus* were slightly higher in the cheese samples of producer A (0.507% and 0.458%, respectively) and producer D (0.993% and 0.892%, respectively). The genus *Lactococcus* was predominant during the cheese making process because of its presence in the starter culture, confirming that bacteria comprising the starter culture grow and predominate, as has been previously observed for Pecorino Siciliano cheese [39]. The proliferation of non-starter LAB (NSLAB) has been reported previously, although there are clear differences according to the type of cheese. For instance, *Lactococcus* and *Lactobacillus* have been reported as predominant in Caciofiore della Sibilla cheese [43], whereas *Lactococcus*, *Carnobacterium* and *Enterococcus* predominate in Canestrato Pugliese cheese [26]. 

In general, the abundance of non-desirable bacteria was less than 1% after cheese making, although the abundance of some bacterial genera, namely *Buttiauxella* (0–5.79%), *Serratia* (0.00179–2.16%) and *Raoultella* (0.0151–1.43%), was maintained at a remarkable level or even increased in cheese obtained from some producers (Figure 1D, Table S2). The opportunistic bacteria *Hafnia*, *Brevibacterium* and *Psychrobacter* [85,86,87], which had low abundance in milk (<1%), also increased their abundances in the cheese of some producers (0.00282–9.62%, 0.0210–2.43% and 0.00168–2.28%, respectively). Notably, these bacterial genera exhibit lipase and/or protease activities [88,89,90], and produce interesting volatile compounds (such as 1-hexanol, 1-propanol, propyl butanoate or butyl butanoate), affecting cheese quality [91,92,93]. Overall, the abundance of environmental bacteria decreased during cheese making, although *Obesumbacterium* maintained a remarkable abundance in samples from producer A (1.89%). Moreover, the environmental genus *Chromohalobacter* [94], which showed low abundance in milk, exhibited higher abundance in cheese, especially that obtained from producer C (1.78%). The abundance of bacteria classified as “others” and of unidentified bacteria in cheese (1.31–2.74% and 1.26–18.6%, respectively) was lower than that in milk (Figure 1D, Table S2). Suppression of the growth of environmental and non-desirable bacteria, such as *Pseudomonas* or *Staphylococcus*, during cheese making has been reported previously [26,43]. Nonetheless, little has been reported about the prevalence of opportunistic or emerging pathogens and environmental bacteria after the cheese making process using raw ewe milk. De Pasquale et al. [26] have detected *Raoultella* in Canestrato Pugliese cheese but not in raw milk and Alegría et al. [32] have reported a prevalence of *Chromohalobacter* in fresh Oscypek cheese. *Hafnia* and *Psychrobacter* have been identified in other cheeses prepared from raw ewe milk [38,95], although the effect of the cheese making process on the abundance of these bacteria is unknown. 

The cheese making process adds other factors that can influence the bacterial communities [9], in addition to factors that determine the milk microbiota (Section 3.2.1). Briefly, the conversion of milk to cheese decreases the pH to 4.5–5.3, which interferes with the growth of most bacteria, except LAB [9,57]. The NaCl concentration of the brine and low salt tolerance of most bacteria only facilitate the growth of LAB [57] and halophiles, such as *Psychrobacter* [96] and *Chromohalobacter* [94]. The decrease in moisture content and water activity (a_w_) also suppresses the proliferation of most bacteria, except LAB, because of their resistance to reduced a_w_ values [9,57]. Moreover, variation that occurs in the redox potential during the conversion of milk to cheese only allows the growth of facultative or obligate anaerobic bacteria [57]. It is worth mentioning that artisanal rennet employed for the production of some raw ewe milk cheeses could be an important source of microorganisms, for example LAB [8]. The use of lamb rennet paste containing pregastric lipase results in higher lipolysis and the development of the characteristic flavour of Idiazabal cheese [97]. Although artisanal rennet contains high levels of a wide range of microorganisms, including aerobic mesophilic bacteria [98], no significant differences have been detected in microbial counts in Idiazabal cheeses prepared using artisanal or commercial rennet [99]; however, it would be interesting to elucidate this aspect using culture-independent methods, such as HTS. Finally, it has been observed that small differences in the environment of dairy facilities producing artisanal cheeses can lead to the development of site-specific “household” microbiota [100]. Therefore, these factors could explain the differences in bacterial composition observed among different raw ewe milk cheeses and among producers producing the same type of cheese.

#### 3.2.3. Bacterial Shifts during the Cheese Ripening Process

Finally, the effect of the ripening process on the bacterial composition of cheese was studied. A total of 23 phyla, 197 families and 583 genera were identified throughout the cheese ripening process; thus, the number of bacterial families and genera was higher during cheese ripening than in raw milk and in cheese after the cheese making process. At the phylum level (Table S1), the abundance of Firmicutes increased (from a mean of 79.4% at 1 day of ripening to 97.7% at 120 days of ripening), while that of Proteobacteria decreased sharply (from 8.56% to 0.116%). In general, abundance of the remaining phyla not predominant during the cheese making process and that of “others” and unidentified bacteria were reduced, except Actinobacteria in the cheese of some producers; nonetheless, the change in abundance levels was not significant for all phyla. Overall, the predominance of Firmicutes and reduction in the abundance of Proteobacteria and remaining phyla have previously been reported in other raw ewe milk cheeses such as Liqvan cheese [38,44]. 

**Figure 2:**
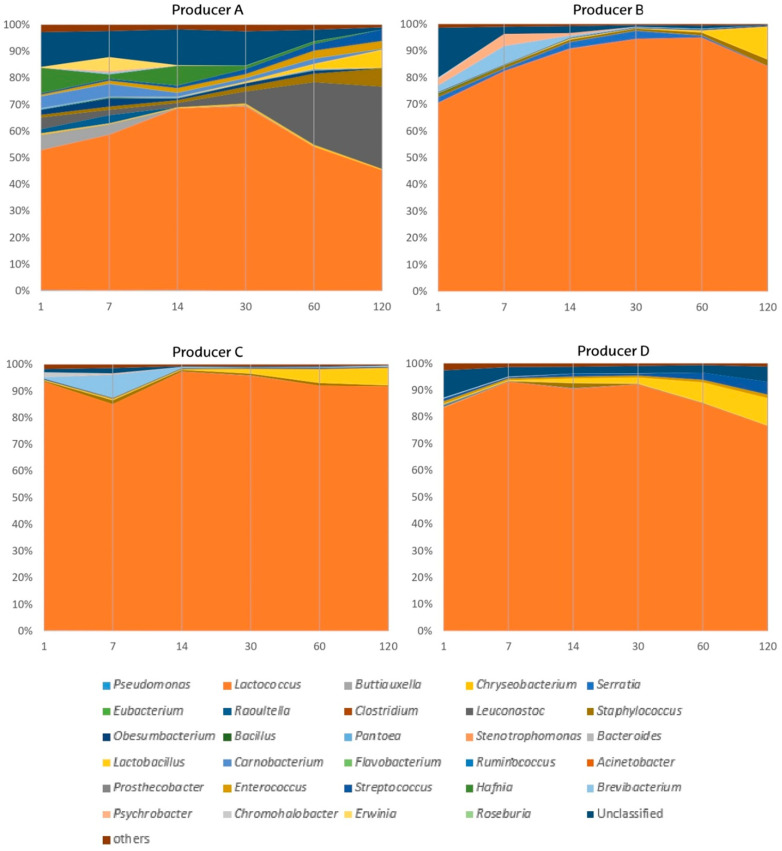
Microbial shifts at genus rank during ripening time (from 1 to 120 days) of Idiazabal cheese samples from different producers (**A**–**D**).

**Figure 3:**
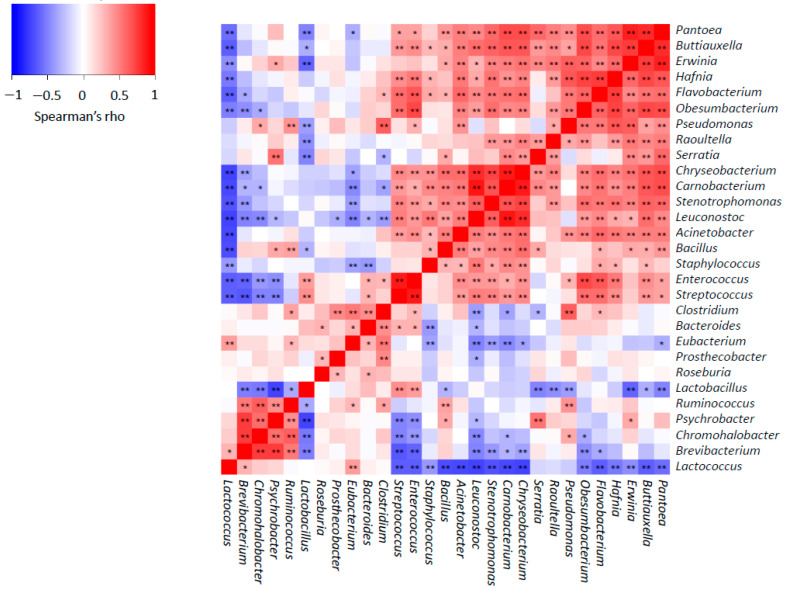
Spearman’s rank correlations between main bacterial genera found in Idiazabal cheese samples. Significant correlations are represented by ** *p* ≤ 0.01 and * *p* ≤ 0.05.

The cheese ripening time had a considerable impact on bacterial abundance at the genus level, resulting in large differences among producers (Figure 2, Table S2). Within LAB, *Lactococcus* remained the most dominant genus in Idiazabal cheese during ripening at all times and for all producers (mean abundance: 74.9% at 1 day of ripening, and 74.5% at 120 days of ripening), except producer A, which showed notably lower proportions of *Lactococcus*. The effect of ripening time on bacterial abundance was significant only for *Lactobacillus*, with an increase in its abundance for all producers (from 0.0949% to 8.96%), while the evolution of the abundance of the remaining genera varied with the producer. In cheeses from producer A, the abundance of *Leuconostoc*, which increased after cheese making, was unquestionably promoted by ripening time (from 4.48% to 31.0%), whereas that of *Carnobacterium* decreased (from 4.40% to 0.330%). In cheeses obtained from producers A and D, the abundances of *Streptococcus* and *Enterococcus*, which increased during the cheese making process, also increased during ripening (from 0.750% to 4.52% and from 0.675% to 2.12%, respectively). Overall, taking into account LAB dynamics (Table S2) and their correlations during ripening time (Figure 3), a clear pattern was observed. The abundance of *Lactococcus* decreased over 30 or 60 days of ripening, depending on the producer, when NSLAB (*Leuconostoc*, *Lactobacillus*, *Streptococcus* and *Enterococcus*) began to proliferate. In other words, from the first ripening month on these NSLAB begin to proliferate and become an important part of the final microbiota of the cheese.

The predominance of bacteria added as part of the starter culture has also been previously reported during the ripening of other raw ewe milk cheeses, such as Pecorino Siciliano cheese [38,39]. However, lactose depletion, salt concentration, and low pH and temperature decrease the viability of starter LAB, and depending on lysis rates, the NSLAB gain importance [101]. The proliferation of *Lactobacillus*, *Leuconostoc*, *Streptococcus* or *Enterococcus* has also been observed in other raw ewe milk cheeses [26,38,39,95]; however, the NSLAB composition of other types of raw ewe milk cheeses is different from that of Idiazabal cheese [38,39,43,44]. These differences are important, since NSLAB affect, among others, the proteolysis and lipolysis of cheese, and consequently, its final properties, including flavour and texture [102,103,104]. 

Overall, the abundance of environmental bacteria, except *Obesumbacterium*, decreased throughout the ripening period; *Obesumbacterium* showed an increase in abundance at 7 days of ripening (3.03%) in samples from producer A (Figure 2, Table S2). Among the non-desirable bacteria, *Hafnia*, *Staphylococcus*, *Buttiauxella*, *Psychrobacter*, *Raoultella*, *Serratia* and *Brevibacterium* remained abundant during cheese ripening (>1%), depending on the producer. Nonetheless, their dynamics differed during ripening. The abundance of *Buttiauxella* decreased throughout the ripening phase, while that of *Staphylococcus* increased until 120 days of ripening. The remaining genera showed an increase in abundance at intermediate time points (at 7, 14 or 30 days of ripening). Moreover, the emerging pathogen *Erwinia* [105], whose abundance was minor in milk (<1%), also showed an increase in abundance in samples from producer A (5.15% at 7 days) (Table S2). Considering that some of these genera, including *Hafnia*, *Brevibacterium* and *Psychrobacter*, are related to the production of volatile compounds [91,92,93], the results of this study suggest that their contribution to the sensory properties of cheese would occur at beginning of the ripening process. The abundance of bacteria classified as “others” and that of unidentified bacteria decreased (from 2.08% to 0.665% and from 10.8% to 1.69%, respectively). Most of these environmental and non-desirable genera have previously been reported in raw ewe milk cheeses [26,37,38,39,43,106,107], including the notable presence of *Staphylococcus* and the increase in its abundances during ripening [26,38,39]. Nonetheless, *Obesumbacterium* and *Hafnia* have only been found in Alberquilla cheese prepared from a mixture of ewe and goat milk [31]. To the best of our knowledge, information on the evolution of most of these bacteria during the ripening period is scarce. 

Furthermore, we examined the correlation among the main bacterial genera during ripening (Figure 3). Spearman’s rank correlations showed some positive relationships between LAB and non-desirable or environmental genera, for example, *Streptococcus*–*Stenotrophomonas*, *Enterococcus*–*Pseudomonas* and *Leuconostoc*–*Bacillus*. However, a remarkable number of negative correlations were detected, confirming that LAB tend to predominate and limit the proliferation of non-desirable or environmental bacteria, as observed previously [44]. Moreover, this supports the idea that the growth of aroma-related bacteria (such as *Hafnia*, *Brevibacterium* and *Psychrobacter*) is inhibited during the first few weeks of ripening [57]. Changes in the physicochemical properties of cheese throughout the ripening process could explain LAB predominance, similar to the cheese making process. Overall, reduced a_w_, high NaCl concentration, refrigeration temperatures during ripening, evolution of oxidation-reduction potential to a more reduced state and the decline in pH may affect the proliferation of most bacteria, and LAB are almost the unique that could proliferate [57]. Moreover, it is well known that competitive interaction mechanisms exist between bacteria [44,108]; for example, LAB produce organic acids or bacteriocins [57,109,110]. However, different parameters, such as temperature and relative humidity, could also affect bacterial proliferation during cheese ripening [57,111] and explain the differentiation observed among the cheeses from different producers.

### 3.3. Overall Effect of Producer and Ripening Time Factors

To examine the effect of producer and ripening time on the main bacterial genera of Idiazabal cheese, a multivariate analysis was performed. PERMANOVA showed that producer and ripening time factors had a statistically significant effect on modulating the microbial composition of Idiazabal cheeses (*p* *≤* 0.001 and *p* *≤* 0.01, respectively) (data not shown), thus confirming the results of univariate analysis (Kruskal-Wallis test). Moreover, the F statistic indicated a higher influence of the producer than that of the ripening time (17.3 and 7.17, respectively) on cheese microbiota. 

**Figure 4:**
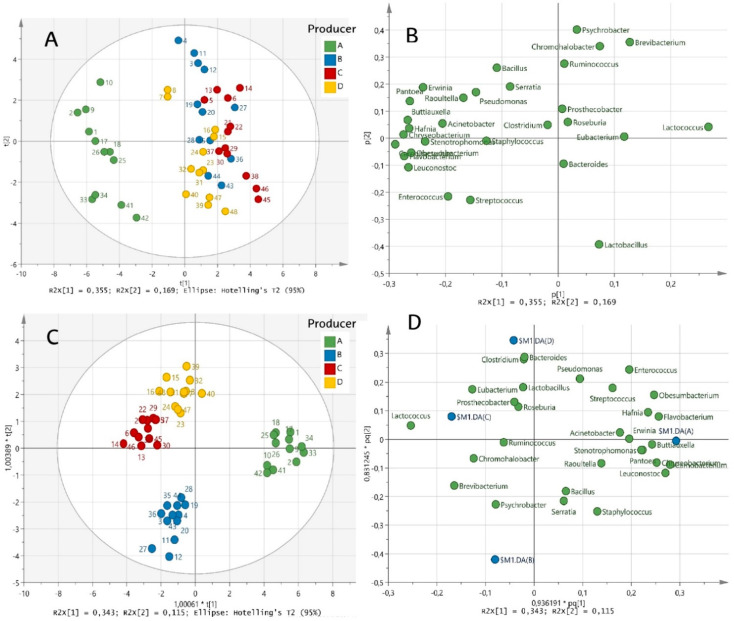
Scores and loadings plots of PCA ((**A**,**B**), respectively) and OPLS-DA ((**C**,**D**), respectively) based on main bacterial genera of Idiazabal cheese samples from four producers (A, B, C and D). Samples are coloured according to the producer and labels indicate samples ID.

PCA of the main bacterial genera identified in cheese samples revealed five PCs (PC1–5), which accounted for 77.0% of the total variance in cheese microbiota due to the producer and ripening time. According to the scores plot (Figure 4A), PC1 (accounting for 35.5% of the explained variance) was related to the producer factor, thus leading to a clear differentiation between the samples of producer A and those of the other producers. According to the loadings plot (Figure 4B), *Lactococcus*, *Carnobacterium*, *Leuconostoc*, *Chryseobacterium*, *Hafnia*, *Buttiauxella*, *Obesumbacterium*, *Pantoea*, *Erwinia*, *Enterococcus*, *Raoultella*, *Streptococcus*, *Pseudomonas* and *Staphylococcus* were highly correlated to PC1 (Figure 4B), indicating that these genera were the most responsible for the differentiation of cheese microbiota among producers. On the other hand, PC2 (accounting for 16.9% of the explained variance) was correlated with the ripening time factor. Therefore, samples were distributed from positive (for less ripened cheeses) to negative (for more ripened cheeses) values (Figure 4A). *Psychrobacter*, *Brevibacterium*, *Chromohalobacter*, *Bacillus* and *Serratia* showed positive loadings in PC2, indicating their disappearance along ripening. Instead, *Lactobacillus* showed negative loadings, indicating that its abundance increased during the ripening phase (Figure 4B). This would confirm the results of the PERMANOVA and indicate that producer factor has a greater impact on cheese microbiota than ripening time.

Compared with PC1 and PC2, the other three PCs, PC3, PC4 and PC5, explained lesser variance in cheese microbiota (11.9%, 6.84% and 5.89% respectively) (Figure S2). Nonetheless, taking together the five PCs provided by the PCA, an idea of the cheeses’ microbiota evolution during the ripening time was obtained for each producer. For producer A, the microbial composition of less ripened cheeses was characterized by *Hafnia*, *Buttiauxella*, *Carnobacterium*, *Obesumbacterium*, *Raoultella*, *Pantoea*, *Chryseobacterium* and *Erwinia* genera. As the ripening progressed, *Lactococcus* proliferated, and the cheese microbiota was finally characterized by high abundance of *Leuconostoc*, *Lactobacillus*, *Streptococcus* and *Enterococcus*. For producer B, less ripened cheeses were characterized by *Serratia*, *Psychrobacter*, *Brevibacterium*, *Chromohalobacter* and *Bacillus*, but as ripening progressed, the microbiota was simplified, with *Lactococcus*, *Lactobacillus* and *Staphylococcus* as the predominating genera. For producer C, less ripened cheeses were characterized by *Chromohalobacter*, *Brevibacterium* and *Pseudomonas*, and throughout ripening, the microbiota was simplified by the predominating genera *Lactococcus* and *Lactobacillus*. For producer D, *Pseudomonas*, *Serratia*, *Bacillus* and *Raoultella* characterized the less ripened cheeses, but the microbiota was predominated by *Lactococcus*, *Lactobacillus*, *Enterococcus* and *Streptococcus* as the ripening progressed. In general, the microbial dynamics described in Section 3.2 were confirmed by multivariate analysis. 

Finally, OPLS-DA, which yielded 3 + 2 + 0 components and with the parameters R2X=0.740 and R2Y=0.898, confirmed the differentiation among producers (Figure 4C). Producer A was clearly distinguished from the other producers, as observed before. However, producer B also showed a clear differentiation from producers C and D. The loadings plot (Figure 4D) revealed the characteristic genera in the cheeses of each producer, corroborating the results of the in-depth analysis of microbial shifts and the PCA.

### 3.4. Alpha and Beta Diversity Analyses

**Figure 5:**
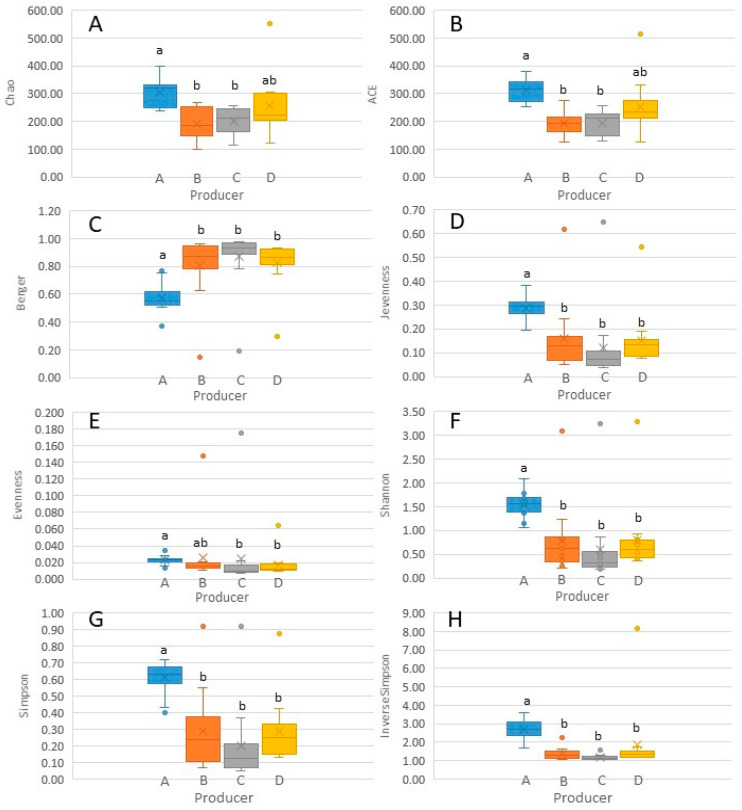
Box plot representation of bacterial alpha diversity indices ((**A**) Chao1; (**B**) ACE; (**C**) Berger; (**D**) Jevenness; (**E**) Eevenness; (**F**) Shannon; (**G**) Simpson; (**H**) Inverse Simpson) of Latxa ewe raw milk and Idiazabal cheese samples obtained from four producers (A, B, C and D). For each diversity index, different letters indicate significant differences between producers at *p* ≤ 0.05.

To analyse alpha diversity, different indices were employed, and the evolution of bacterial richness, evenness and biodiversity was examined (Table S3). Chao1 and ACE richness estimators showed a negative trend during the transition from milk to 120-day-old ripened cheese, which was either more pronounced or less pronounced depending on the producer. This implies that a non-negligible number of bacterial genera originally present in raw ewe milk disappeared during cheese making and ripening. Overall, these results are consistent with what has been previously observed for other raw ewe milk cheeses, such as Liqvan cheese [43,44]. According to the uniformity, Shannon evenness and Berger indices showed a decreasing trend throughout the cheese making and ripening processes, indicating that the microbial population of cheese was dominated by a few genera. Nevertheless, uniformity increased after 30 or 60 days, depending on the producer, since other genera gained importance. To the best of our knowledge, there has been no report to date on the shifts in bacterial uniformity during production and ripening of raw ewe milk-derived cheeses. Finally, combining the measurement of the number of genera and their abundance, the Shannon, Simpson and Inverse Simpson biodiversity indices confirmed a downward trend from milk to 30- or 60-day-old ripened cheeses, depending on the producer. However, subsequently, biodiversity increased until 120 days of ripening. In other words, it was confirmed that up to the first or second month of ripening, a few bacterial genera predominated; however, subsequently, other bacteria proliferated and acquired importance. Ramezani et al. [44] have also reported a greater complexity of biodiversity in raw milk than in curd or cheese, and an increase in biodiversity during the conversion of curd into Liqvan cheese. However, De Pasquale et al. [26] have reported a higher biodiversity in curd after moulding than in milk or final cheese. In general, statistically significant differences were observed in alpha diversity between producer A and the others (Figure 5). Differences in alpha diversity among producers of other raw ewe milk and cheeses have rarely been studied [37].

**Figure 6:**
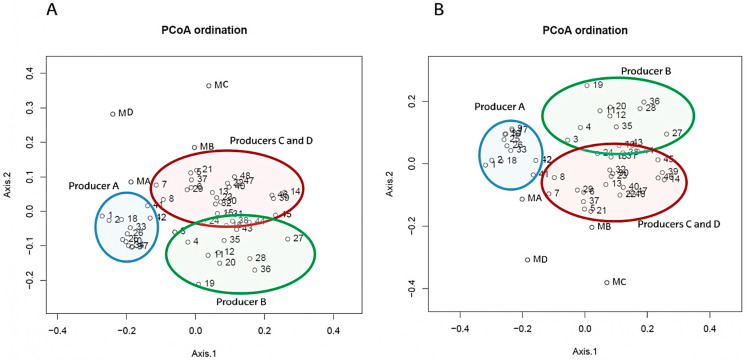
PCoA of bacterial beta diversity at genus rank based on Bray-Curtis (**A**) and Jaccard (**B**) dissimilarities.

Subsequently, to clarify differences in the microbial composition of cheeses among producers, beta diversity was calculated. At genus level, cheese samples were distributed into three clusters corresponding to cheeses from producer A, producer B and producers C and D, which were very similar (Figure 6). Samples from producer A were tightly clustered, indicating less microbial changes during the ripening time compared with cheeses from other producers. Samples collected from producers A and B at 120 days of ripening grouped close to those collected from producers C and D at the same time point, indicating similar bacterial composition among cheese samples of different producers at the end of ripening. In addition, milk samples were far from the general dispersion of cheese samples, indicating clear differences in bacterial composition between the two sample types. To the best of our knowledge, very few studies have been published comparing beta diversity of the same raw ewe milk and cheese among different producers [37,39,40,44]. Endres et al. [40] have reported differences in raw ewe milk samples among different dairies, and Cardinali et al. [37] have reported differences among the producers of Queijo de Azeitão cheese. Beta diversity has also been used to differentiate among the different types of ewe cheeses [95] and to analyse the effect of specific starters on cheese microbiota [39].

Taking together, alpha and beta diversity indices confirmed the results of the in-depth analysis of microbial shifts, univariate analysis (Kruskal-Wallis test) and multivariate analyses (PERMANOVA, PCA and OPLS-DA). The cheese making and ripening processes had an undoubted impact on the bacterial communities. Overall, bacteria from the starter culture predominated at the beginning of ripening, but after 30 or 60 days of ripening, the bacteria from raw milk, especially NSLAB, began to proliferate and become noticeable. Nonetheless, clear differences in the microbial composition of raw ewe milk and cheese samples were observed among producers, which could indicate that differences in practices, such as flock management and milking, as well as parameters selected during cheese making and ripening processes would determine the final microbiota.

## 4. Conclusions

This is the first HTS study carried out with the objective of characterizing the microbiota of Latxa ewe raw milk and examining the bacterial shifts that occur during the production and ripening of Idiazabal cheese. This research confirms that HTS techniques allow a better understanding of the microbial communities, which could not be achieved previously using culture-dependent techniques. Several bacterial genera were detected for the first time in raw ewe milk and cheese. Both the cheese making process and ripening time had a remarkable impact on bacterial communities, although considerable differences were observed among producers. Thus, the use of raw milk and the practices and conditions employed by each producer for flock management, milking and cheese making and ripening could determine the microbiota. The growth of LAB was promoted throughout the cheese making and ripening processes, whereas that of non-desirable and environmental bacteria was inhibited. However, LAB composition differed among producers, and the growth of NSLAB was promoted after 30 or 60 days of ripening. In addition, in some cases, bacteria related to the production of volatile compounds (such as *Hafnia*, *Brevibacterium* and *Psychrobacter*) showed notable abundance during the first few weeks of ripening.

## Figures and Tables

**Table 1:** Metataxonomic data of Latxa ewe raw milk and Idiazabal cheese samples at 6 ripening times (1, 7, 14, 30, 60 and 120 days) from 4 producers (A, B, C and D) (*n* = 52).

Producer	Milk/Cheese Ripening Time (Day)	Sample ID	Bacterial Diversity					
			Sequences	OTUs	Mbp Count	Phyla	Families	Genera
A	Milk	MA	66,989	8450	2.70	8	103	221
	1	1	321,148	21,102	6.84	10	102	244
		2	331,816	17,948	5.96	10	107	242
	7	9	238,804	12,412	4.21	10	82	182
		10	251,225	13,472	4.55	10	92	203
	14	17	263,231	13,305	4.51	10	81	176
		18	334,305	16,504	5.95	11	90	209
	30	25	269,207	14,266	5.18	8	89	195
		26	280,285	13,701	4.65	8	84	191
	60	33	330,729	15,810	5.50	10	79	179
		34	341,419	16,324	5.92	10	79	183
	120	41	370,181	22,557	7.61	14	83	187
		42	332,471	16,882	6.15	12	78	170
B	Milk	MB	6092	2504	0.769	10	66	136
	1	3	257,407	12,967	4.39	9	90	178
		4	184,670	10,745	3.66	7	80	158
	7	11	160,436	9174	3.15	7	70	124
		12	148,700	8429	3.09	9	67	117
	14	19	193,164	8672	2.94	9	57	107
		20	134,695	7270	2.48	7	55	107
	30	27	140,484	2919	1.31	7	40	60
		28	129,491	6422	2.33	6	43	88
	60	35	295,377	13,046	4.38	10	64	125
		36	157,505	7103	2.46	6	47	91
	120	43	294,909	12,781	4.61	10	54	107
		44	175,689	8629	3.13	10	53	95
C	Milk	MC	10,632	4889	1.41	10	66	135
	1	5	237,563	11,167	3.76	10	83	165
		6	200,211	9565	3.24	10	73	156
	7	13	172,573	9232	3.16	9	72	136
		14	105,377	2947	1.32	5	39	65
	14	21	281,503	11,879	4.03	10	76	152
		22	162,633	7438	2.54	8	57	112
	30	29	374,652	14,534	5.20	8	75	164
		30	178,935	7641	2.65	9	66	125
	60	37	390,610	14,658	5.06	12	83	170
		38	218,602	8265	2.84	8	57	105
	120	45	116,160	5909	2.05	5	47	76
		46	188,779	8633	2.99	7	51	83
D	Milk	MD	52,040	11,547	3.80	21	151	378
	1	7	210,147	11,128	3.71	10	97	217
		8	175,444	8825	3.03	11	91	197
	7	15	136,796	6512	2.38	7	65	120
		16	126,129	6062	2.11	8	56	113
	14	23	194,767	8284	2.99	11	66	131
		24	201,259	9138	3.09	14	73	139
	30	31	161,519	7050	2.39	13	68	119
		32	187,889	8164	2.78	10	66	136
	60	39	86,466	4525	1.68	8	45	84
		40	202,656	8933	3.28	7	65	127
	120	47	205,505	9667	3.36	11	67	119
		48	209,716	10,185	3.51	15	63	123

## Data Availability

The data are not publicly available yet as some data sets are being used for additional publications.

## Supplementary Materials

The following supporting information can be downloaded at: https://www.mdpi.com/article/10.3390/biology11050769/s1, Figure S1: Rarefaction curves of microbial populations of the studied samples from each producer. Each graph represents a producer (A, B, C and D) and each line is coloured according to the Sample ID; Figure S2: Scores and loadings plots of PCA based on main bacterial genera of Idiazabal cheeses from 4 producers (A, B, C and D). Samples are coloured according to the producer and labels indicate samples identification; Table S1: Mean and standard deviation of bacterial phyla of Latxa ewe raw milk and Idiazabal cheese samples at 6 ripening times (1, 7, 14, 30, 60 and 120 days) from 4 producers (A, B, C and D) (*n* = 52).; Table S2: Mean and standard deviation of bacterial genera of Latxa ewe raw milk and Idiazabal cheese samples at 6 ripening times (1, 7, 14, 30, 60 and 120 days) from 4 producers (A, B, C and D) (*n* = 52): Table S3: α-diversity indices of Latxa ewe raw milk and Idiazabal cheese samples at 6 ripening times (1, 7, 14, 30, 60 and 120 days) from 4 producers (A, B, C and D) (*n* = 52).
