# Supplementary material for: Characterization of Microbial Shifts during the Production and Ripening of Raw Ewe Milk-Derived Idiazabal Cheese by High-Throughput Sequencing
Source: Biology (Basel). 2022 May 18;11(5):769. doi: 10.3390/biology11050769 (PMC9138791; doi:10.3390/biology11050769)
Supplement: Supplementary file 1 [file biology-11-00769-s001.zip › Figure S1.pdf]

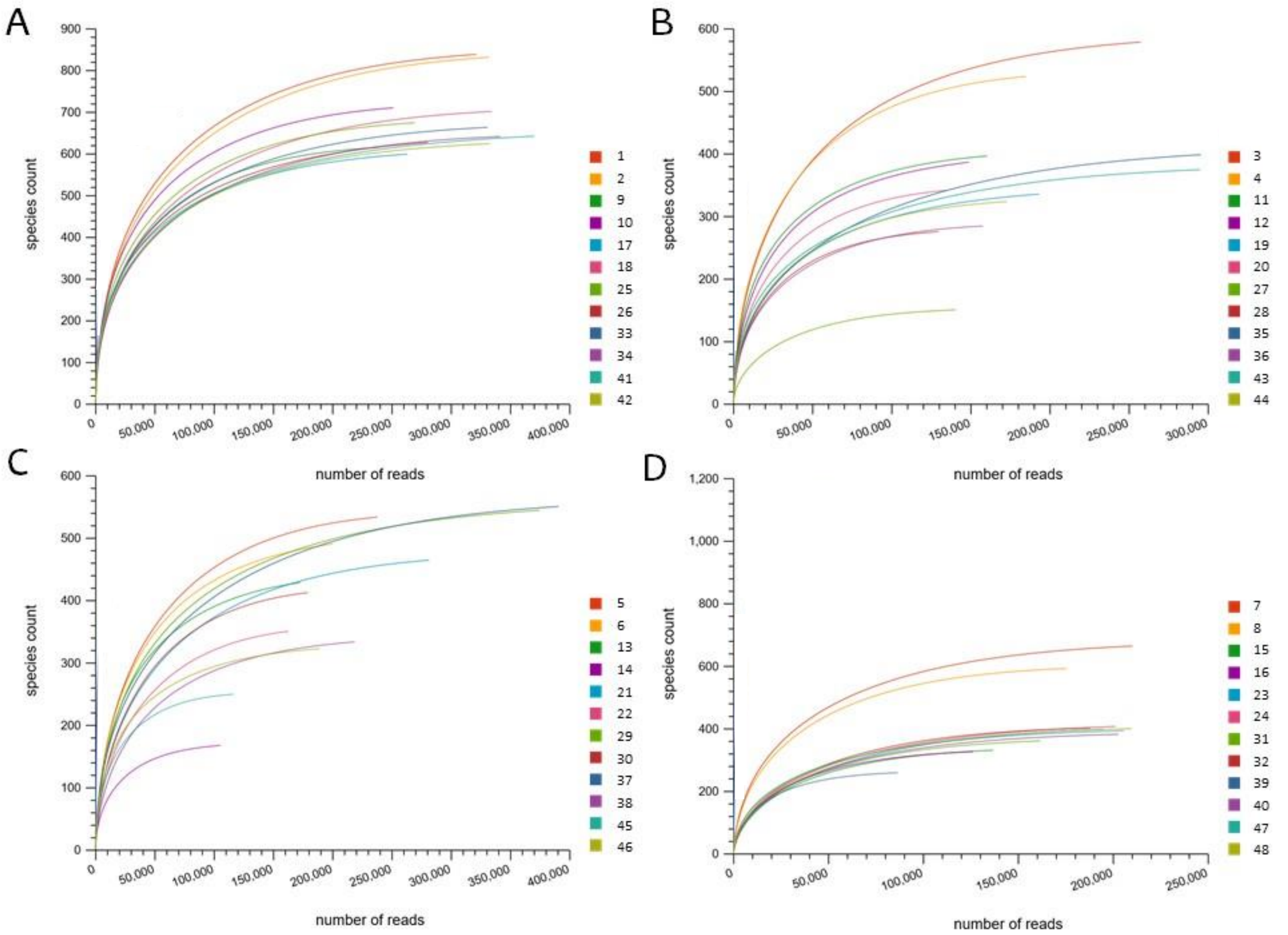

Figure S1. Rarefaction curves of microbial populations of the studied samples from each producer. Each graph represents a producer (A, B, C and D) and each line is colored according to the Sample ID.
