# Supplementary material for: Characterization of Microbial Shifts during the Production and Ripening of Raw Ewe Milk-Derived Idiazabal Cheese by High-Throughput Sequencing
Source: Biology (Basel). 2022 May 18;11(5):769. doi: 10.3390/biology11050769 (PMC9138791; doi:10.3390/biology11050769)
Supplement: Supplementary file 1 [file biology-11-00769-s001.zip › Figure S2.pdf]

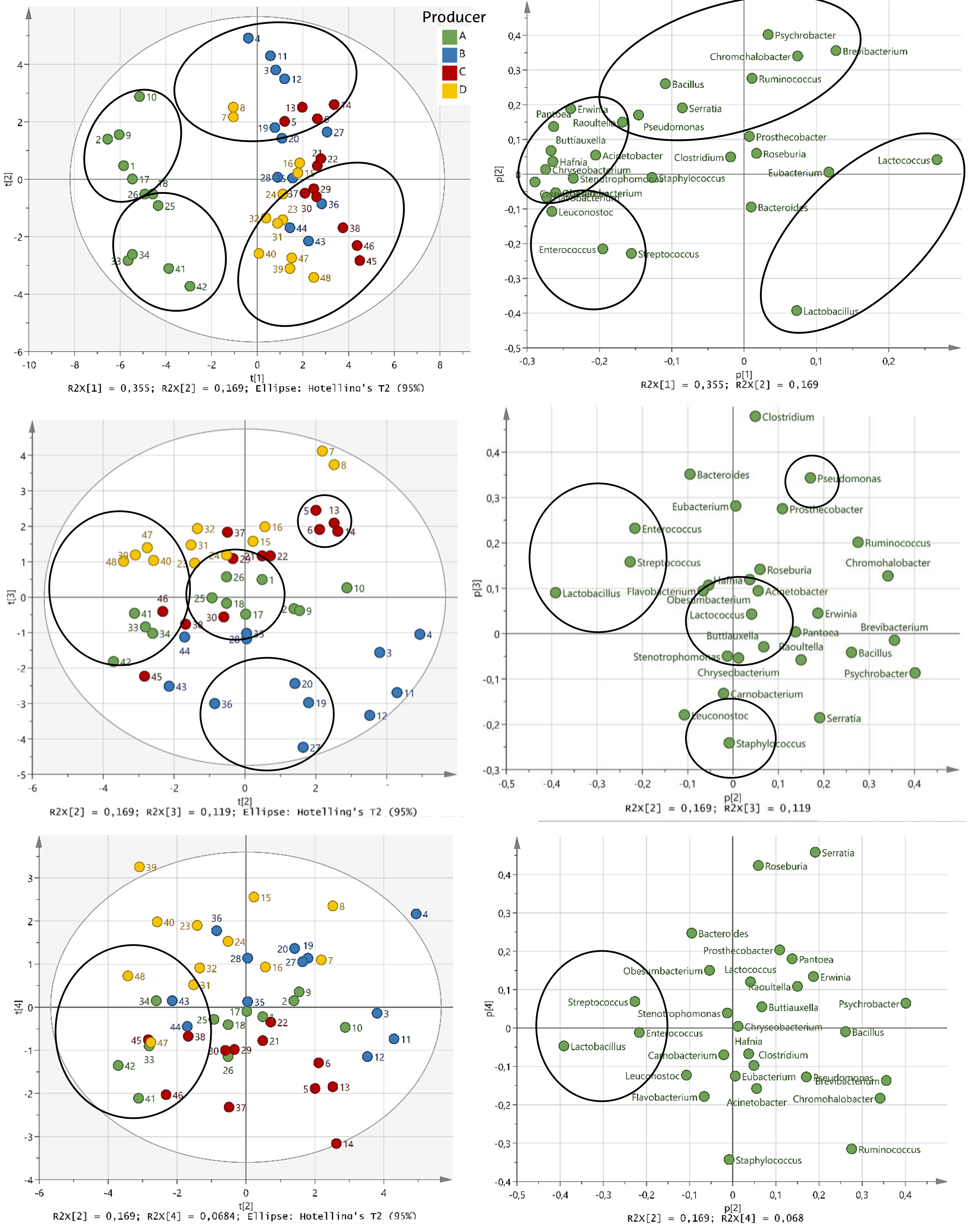

Figure S2. Scores and loadings plots of PCA based on main bacterial genera of Idiazabal cheeses from 4 producers (A, B, C and D). Samples are colored according to the producer and labels indicate samples identification.
